# The Safe Baculovirus-Based PrM/E DNA Vaccine Protected Fetuses against Zika Virus in A129 Mice

**DOI:** 10.3390/vaccines9050438

**Published:** 2021-04-30

**Authors:** Hanul Choi, Jungmin Chun, Mina Park, Suyeon Kim, Nahyun Kim, Hee-Jung Lee, Minjee Kim, Ha Youn Shin, Yu-Kyoung Oh, Young Bong Kim

**Affiliations:** 1 Department of Bioindustrial Technologies, Konkuk University, Seoul 05029, Korea; chlgksmf9977@hanmail.net; 2 Center for Glocal Disease Control, KR BioTech, Seoul 05029, Korea; anananq@naver.com; 3 Department of Biomedical Science and Engineering, Konkuk University, Seoul 05029, Korea; min.a@outlook.kr (M.P.); 0924tndus123@naver.com (S.K.); knh64@naver.com (N.K.); ziniga@konkuk.ac.kr (H.-J.L.); mj0411@konkuk.ac.kr (M.K.); hayounshin@konkuk.ac.kr (H.Y.S.); 4 College of Pharmacy and Research Institute of Pharmaceutical Sciences, Seoul National University, Gwanak-gu, Seoul 08826, Korea; ohyk@snu.ac.kr

**Keywords:** vaccine, ZIKV, baculovirus system, arbovirus, nonreplicated viral vector

## Abstract

The Zika virus (ZIKV) is a mosquito-borne member of the *Flaviviridae* family of enveloped RNA viruses. The correlation between viral infection and fetal microcephaly was revealed in 2015, yet we still lack a vaccine against ZIKV. Here, we present a genetic vaccine that delivers the premembrane (prM) and envelope (E) genes of ZIKV using a recombinant baculovirus vector that expresses a human endogenous retrovirus (HERV) envelope on its surface to enhance gene delivery. We observed that baculoviruses with HERV envelopes (AcHERV) exhibited specifically higher gene transfer efficiency in human cells compared to the wild-type baculovirus vector. Using the AcHERV baculovirus vector, we constructed a recombinant baculovirus vaccine encoding ZIKV prM/E genes (AcHERV-ZIKV), which are major targets of neutralizing antibodies. Mice immunized twice with AcHERV-ZIKV exhibited high levels of IgG, neutralizing antibodies, and IFN-γ. In challenge tests in IFN knock-out mice (A129), AcHERV-ZIKV showed complete protection in both challenge and pregnancy tests. These results suggest that AcHERV-ZIKV could be a potential vaccine candidate for human application.

## 1. Introduction

The Zika virus (ZIKV) is a mosquito-borne flavivirus that has a single-strand RNA genome [1,2]. The ZIKV RNA genome encodes three structural proteins (capsid, prM, and envelope) and seven nonstructural proteins (NS1, NS2A, NS2B, NS3, NS4A, NS4B, and NS5) [3]. ZIKV was first identified in 1947 from a monkey in the Zika Forest of Uganda; the strains are classified into two lineages, African and Asian/American [1,2]. An outbreak of ZIKV occurred in Brazil in 2015, with nearly 30,000 cases reported [4]. Brazil also experienced a sharp rise in the number of cases of pregnancy-associated microcephaly, which was later discovered to be linked to ZIKV infection [5,6]. *Aedes aegypti*, which is the most common mosquito species infiltrating the cities of Brazil, served as a main virus vector to spread the disease [7]. In 2016, the World Health Organization (WHO) declared the current ZIKV outbreak as a public health emergency of international concern [7]. At this time, there is still no licensed vaccine or treatment available. Therefore, vaccine development is an urgent and primary goal to prevent ZIKV infection and provide protection against congenital anomalies of infection during pregnancy [8].

Since prM/E protein is located in the outermost part of the Zika virus and has the highest antigenicity, it is the main target of antibody–antibody reaction, and it has been selected as a target antigen in several studies, and its antigenicity for vaccine development has been sufficiently proven [9,10,11]. It also mediates viral assembly and adhesion to cellular receptors, such as other flaviviruses, and is an essential protein for subsequent membrane fusion related to viral entry. Therefore, we selected the prM/E gene of Zika virus as the target gene [9,10].

Several ZIKV vaccines have been studied and moved toward clinical trial, including those based on virus like particles (VLP), nucleic acid vaccines, purified inactivated virus, vector-based vaccines, and live-attenuated ZIKV [8]. A plasmid DNA vaccine encoding the prM and E gene sequences was able to completely protect mice and rhesus monkeys against ZIKV challenge [12]. However, it seems relevant that in the development of the SARS-CoV2 vaccine, the mRNA vaccine successfully passed clinical trials while the DNA vaccine had poor clinical results. This is because the DNA plasmid works only when it enters the nucleus, which limits its practicality for clinical application. Viral vectors are a favored method in vaccine development [13]. Viruses represent the best gene delivery systems and viral vectors can provide a convenient means to deliver vaccine antigens to select target cells or tissues [14]. In the context of ZIKV, adenovirus vector vaccines encoding the prM/E genes have successfully protected ZIKV in animal models, and vaccinia virus vectors have also been tested [15,16,17,18,19]. For clinical applications, however, adenovirus and vaccinia viral vectors suffer from limitations associated with preexisting immunity, cytotoxicity, and undesired gene expression from the viral vector [20]. In general, mammalian viral vectors can be problematic for use in vaccines that require multiple immunizations.

Our research team proposes a new platform: a baculovirus vector vaccine with a human endogenous retrovirus (HERV) envelope. The Baculovirus with HERV *env* (AcHERV) system offers several advantages as a DNA vaccine. One major advantage is safety because, compared to other viral vectors, baculoviral genes are mostly silent in mammals [21]. Another advantage of the AcHERV system is the enhanced cellular uptake of AcHERV due to the presence of HERV envelope proteins on the virus surface. Our AcHERV system efficiently delivers vaccine genes into human cells through type D retrovirus receptor (RDR) binding-dependent endocytosis with multiple boosting [22].

Here, we constructed ZIKV prM/E gene-delivering AcHERV baculoviruses and evaluated their immunogenicity.

## 2. Materials and Methods

### 2.1. Cells and Viruses

African green monkey kidney cells (Vero cells) and human embryonic kidney (HEK) 239TT cells were cultured in Dulbecco’s Modified Eagle’s Medium (DMEM) supplem ()ented with 10% fetal bovine serum (Gibco BRL, CA, USA) at 37 °C in a 5% CO_2_ incubator. Spodoptera frugiperda 9 (Sf9) (Invitrogen, USA) cells were maintained in Sf-900 medium with 3% fetal bovine serum (Gibco BRL, US) at 27 °C. African strain MR766 (Accession: AY632535) and Asian strain FLR (Accession: KU820897) were obtained from ATCC (VA, USA).

### 2.2. Plasmid Construction and Gene Expression

The partial gene of HERV envelope was subcloned into the recombinant baculovirus vector, pFastBac1 (Invitrogen, CA, USA). The prM/E gene of ZIKV was synthesized from the Asian strain (Gene Art, MA, USA), and the signal sequence was replaced with that of various sources. The modified ZIKV prM/E gene was subcloned into the pcDNA3.1(+) vector, PCR amplified from the Cytomegalovirus (CMV) promoter to the BGH tail, and introduced into the PstI site in pFastBac-HERV. Recombinant baculovirus was produced using the Bac-to-Bac baculovirus expression system (Invitrogen, USA) according to the manufacturer’s protocol and was titrated by qRT-PCR analysis. 239TT cells were seeded to a 6-well plate and incubated at 37 °C in 5% CO_2_. After 24 h, cells were infected with recombinant baculovirus at 10 MOI. Three days after infection, 293TT cells were harvested, cell lysates were resolved by 10% SDS-PAGE, proteins were transferred to a nitrocellulose membrane, and the membrane was incubated with anti-ZIKV envelope antibody (Genetex, CA, USA). The expression of b-actin was detected as a loading control using an anti-b-actin antibody (Santa Cruz Biotech, TX, USA). HRP-conjugated monoclonal goat anti-mouse antibody was used as a secondary antibody (Abcam, Cambridge, UK).

### 2.3. Mice

C57BL/6 mice were obtained from Orient Bio (Gyeonggi, Korea) and maintained for one week. Interferon alpha/beta (IFN-α/β) receptor-deficient A129 mice were obtained from the Korea Research Institute of Chemical Technology (KRICT, Daejeon, Korea). Groups of 6- and 8-week-old mice were used for experiments. All animal experiments were performed in BL2 animal facilities according to the relevant guidelines, and the laboratory procedures were approved by the Konkuk University Institutional Animal Care and Use Committee (IACUC approval number: KU19213).

### 2.4. Enzyme Linked Immunosorbent Assay (ELISA)

Levels of ZIKV-specific antibodies were measured in vaccinated mice using indirect ELISA. Briefly, an Immuno 96-well plate (Thermo Fisher, MA, USA) was coated with ZIKV MR766 (5 × 10^2^ PFU/well) in carbonate-bicarbonate buffer (pH 9.6) and incubated at 4 °C overnight. The plate was blocked with 5% skim milk in Phosphate-buffered saline (PBS, Biosesang, Gyeonggi, Korea) for 1 h at 37 °C and each well was washed with PBS containing 0.05% Tween 20. Mouse sera were serially diluted with PBS and plated to the wells, and the plate was incubated for 3 h at room temperature. The wells were washed three times with PBS containing 0.05% Tween 20, HRP-conjugated monoclonal goat anti-mouse secondary antibody (Abcam, Cambridge, UK) was added, and the plate was incubated for 1 h at 37 °C. TMB substrate (Invitrogen, CA, USA) was added to each well and the reaction was stopped by the addition of 1N H_2_SO_4_. The absorbance at 450 nm was determined by a microplate reader. The cut-off (endpoint titer) was determined based on the negative control group serum titer and standard deviation. The reciprocal of the penultimate serum dilution above the cut-off was taken as the antibody titer.

### 2.5. Enzyme-Linked ImmunoSpot (ELISPOT) Assay

The levels of interferon gamma (IFN-γ) produced by the splenocytes of immunized mice were detected by an ELISPOT assay. A 96-well plate was coated with 0.2 µg of anti-mouse capture antibody and blocked for 1 h with RPMI-1640 medium at room temperature. Splenocytes (1 × 10^6^/well) were seeded and incubated with 5 × 10^4^ PFU ZIKV MR766 strain as a stimulating antigen. After 24 h, the plate was washed with deionized water and PBS containing 0.05% Tween 20, and treated with 20 µg of biotinylated anti-mouse IFN detection antibody. After 2 h of incubation, streptavidin-alkaline phosphatase was added, and the plate was incubated for 1 h. Spots were developed using an AEC substrate reagent (BD Bioscience, NJ, USA).

### 2.6. Neutralizing Antibody Assay

Neutralizing antibody titers were estimated by a 50% plaque reduction neutralization test (PRNT50). To measure ZIKV Neutralizing antibody titers, approximately 50 PFU of ZIKV (MR766) was mixed with pooled and serially diluted serum samples obtained from the various immunization groups. The samples were incubated for 1 h at 37 °C, added to monolayers of Vero cells in 6-well plates, overlaid with 1.5% agar and an equal volume of DMEM, and incubated for 5 days at 37 °C. The plates were then washed, stained with crystal violet, and dried for plaque counting. Percent neutralization was calculated by comparison to samples containing the same dilutions of control serum from unimmunized animals.

### 2.7. ZIKV Challenge in A129 Mice

A129 mice were used for the vaccine efficacy tests and ZIKV MR 766 strain was used for the virus challenge. The study tested the efficacy of two doses of recombinant baculovirus vaccine in 8-week-old female A129 mice (*n* = 7/group) against challenge with ZIKV. The vaccine group was intramuscularly vaccinated on day 0 and day 21 with a dose of 5 × 10^7^ FFU (focus forming unit) and the control group received equivalent doses of placebo (AcHERV-eGFP). Prior to virus challenge, all mice were assessed for ZIKV-specific IgG and neutralizing antibody titers. Mice were challenged with 10^4^ PFU of ZIKV MR766 via the intraperitoneal (IP) route on day 28. On day 3 post-infection, sera were isolated from all mice of each group; the sera were assessed for viremia by real time PCR. All groups were monitored daily for weight loss and mortality for 9 days post-challenge or until the time of sacrifice. Mice showing lethargy, paralysis, and/or weight loss of greater than 20% were euthanized. After all the mice in the control group died, the mice in the vaccine group were euthanized and the virus titers of the organs were measured by qRT-PCR.

### 2.8. Virus Infectivity Test in Pregnant Mice

The vaccine group was intramuscularly vaccinated with doses of 5 × 10^7^ FFU on days 0 and 21, and the control group received an equivalent dose of placebo (AcHERV-eGFP). The vaccinated mice were mated with male C57BL/6 mice on the 28th day post-vaccination. Based on vaginal plug check, mice were challenged on day E5.5 with 5 × 10^4^ PFU of ZIKV FLR through the IP route. Samples of fetus and placenta were harvested on day E15.5 and viral loads were measured by qRT-PCR (Takara Bio, CA, USA) analysis.

### 2.9. Data Analysis

All data were analyzed using the GraphPad Prism6 software (GraphPad Software Inc., CA, USA). A value of *p* < 0.05 was considered statistically significant. Significance of the survival rates was assessed by survival curve test. The morphological measurements were assessed by one- or two-way Analysis of Variance (ANOVA).

## 3. Results

### 3.1. Construction and Assessment of ZIKV PrM/E Protein-Expressing Recombinant Baculoviruses Regulated by Different Signal Peptides

**Figure 1:**
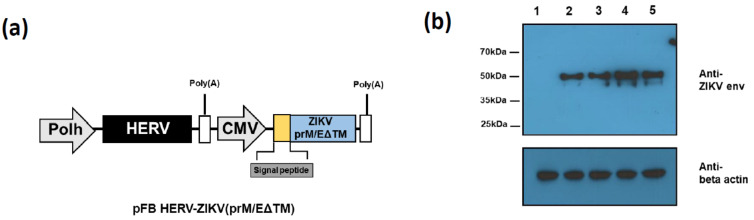
Generation of ZIKV prM/E-expressing baculovirus vectors regulated by different signal peptides. (**a**) Schematic diagram of recombinant ZIKV prM/E gene-delivering baculovirus vectors. (**b**) Expression levels of ZIKV prM/E controlled by various signal peptides. 293TT cells were infected with ZIKV prM/E-expressing baculoviruses regulated by signal sequences (SS) from different origins. Expression levels of prM/E were detected by Western blot analysis using anti-ZIKV ENV antibody. Lane 1, NTC; lane 2, AcHERV-ZIKV (prM/EΔTM with ZIKV SS); lane 3, AcHERV-ZIKV (CD5 SS-prM/EΔTM); lane 4, AcHERV-ZIKV (IgK_SS-prM/EΔTM); lane 5, AcHERV-ZIKV (IgM_SS-prM/EΔTM).

The first priority when developing a DNA vaccine is the optimization of antigen gene expression. For the development of a baculovirus vector-mediated ZIKV vaccine, we synthesized a codon-optimized prM/E gene from the Asian ZIKV strain. To enhance ZIKV prM/E gene expression, we replaced the signal sequence (SS) of ZIKV prM/E with those of human CD5, IgK, or IgM (Table 1). We further removed the transmembrane domain to promote immune response [18]. The obtained recombinant plasmids were cloned into the pFastBac-HERV vector. The generated four types of baculovirus were compared expression intensity through Western blot analysis after 293TT infected. AcHERV-ZIKV (prM/EΔTM with ZIKV SS) and AcHERV-ZIKV (CD5 SS-prM/EΔTM) showed similar expression levels, while AcHERV-ZIKV (IgK SS prM/EΔTM) and AcHERV-ZIKV (IgM SS prM/EΔTM) showed higher expression levels than AcHERV-ZIKV (prM/EΔTM) (Figure 1b).

### 3.2. Recombinant ZIKV PrM/E-Expressing Baculoviruses Successfully Induce ZIKV-Specific Antibodies and T-Cell Responses

**Figure 2:**
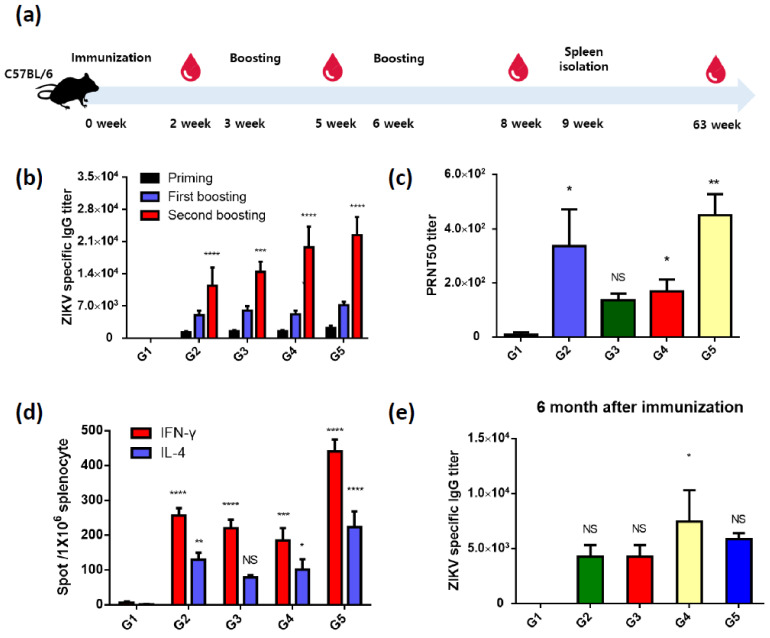
Immunogenicity in C57BL/6 mice vaccinated with ZIKV prM/E-expressing baculoviruses. (**a**) Schematic depiction of vaccination procedures in C57BL/6 mice. (**b**) Humoral immune responses were evaluated by detecting the ZIKV-specific IgG titer using indirect ELISA. (**c**) Generation of ZIKV-specific neutralizing antibodies was assessed by calculating the PRNT50 using immunized mouse sera. Mouse sera obtained after the 2nd boosting were analyzed. (**d**) T-cell-mediated immune responses were examined by IFN-γ and IL-4 ELISPOT analyses. Splenocytes isolated from vaccinated mice were stimulated with 1 × 10^5^ PFU ZIKV MR766 strain. (**e**) The long-term immune memory response was assessed after 6 months of vaccination. **** *p* < 0.0001, *** *p* < 0.001, ** *p* < 0.01, * *p* < 0.05, NS, not significant, compared with the PBS group.

To evaluate the ZIKV immune response after vaccination, 6-week-old female C57BL/6 mice were vaccinated with 4 × 10^7^ FFU of recombinant baculovirus by intramuscular (IM) injection, with 3-time injections given at 3-week intervals. Blood samples were collected 1 week before boosting, and mice were sacrificed 1 week after the final boosting (Figure 2a and Table S1). (G1: PBS; G2: AcHERV-ZIKV (prM/EΔTM with ZIKV SS); G3: AcHERV-ZIKV (CD5 SS prM/EΔTM); G4: AcHERV-ZIKV (IgM SS prM/EΔTM); G5: AcHERV-ZIKV (IgM SS prM/EΔTM)) All immunized groups showed high titers of anti-ZIKV IgG (G2: 11,400 ± 8848.73; G3: 14,400 ± 4929.50; G4: 19,800 ± 9859.01; G5: 22,400 ± 8763.56 means ± SEM) (Figure 2b) and anti-ZIKV neutralizing antibodies (Figure 2c). Our neutralizing antibody analysis of sera from five mice per group revealed that G2 showed high titers in two of the five mice while G5 showed evenly high neutralizing antibody titers in all five analyzed mice.

In our assessment of T-cell responses using an ELISPOT assay, all immunized groups showed high levels of IFN and IL-4, with the IgM group (G5) showing the highest responses (Figure 2d). To compare the duration of immunity, sera were isolated from 3 mice of each group at 6 months post-vaccination (Figure 2e). At this time, the antibodies titer persisted above 5000 in all groups. The AcHERV-ZIKV (IgM_SS-prM/EΔTM)-vaccinated group showed the strongest B-cell and T-cell responses, we decided to use AcHERV-ZIKV (IgM_SS-prM/EΔTM) as a potential vaccine candidate for further study. Below, it is called AcHERV-ZIKV.

### 3.3. Mice Vaccinated with AcHERV-ZIKV Are Fully Protected against ZIKV Infection

**Figure 3:**
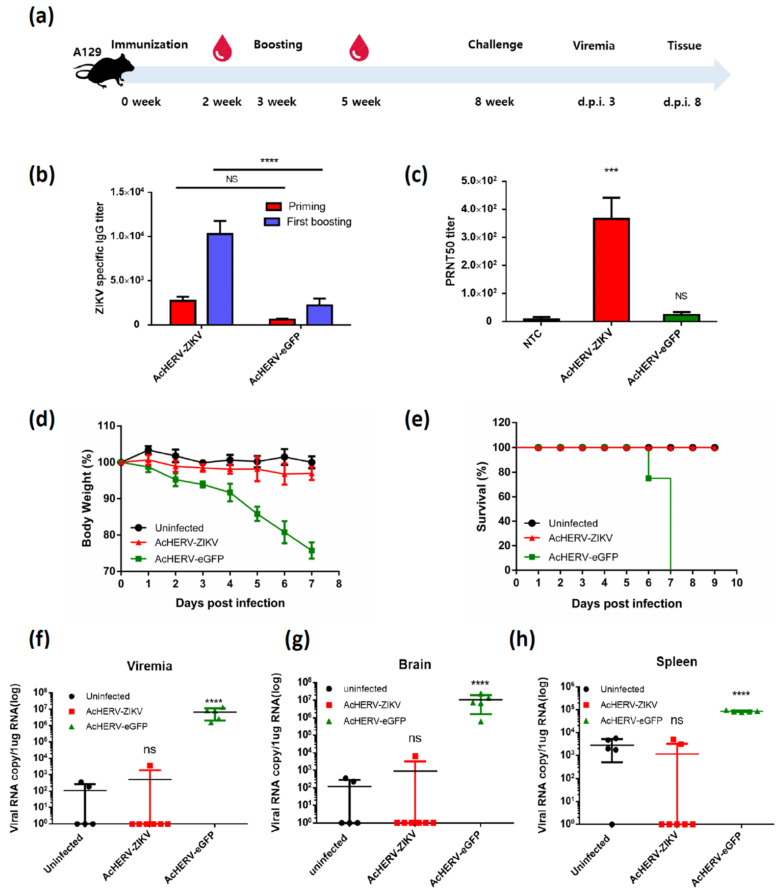
Assessment of vaccine efficacy in ZIKV-challenged A129 mice. (**a**) Experimental design used to assess the effect of vaccination in ZIKV-challenged A129 mice. (**b**) Humoral immune responses were evaluated using indirect ELISA. (**c**) Levels of ZIKV-specific neutralizing antibodies were analyzed using immunized mouse sera. **** *p* < 0.0001, *** *p* < 0.001, ns, not significant, compared with the placebo group. (**d**) Survival rates of mice after the ZIKV challenge. (**e**) Loss of body weight was analyzed after the ZIKV challenge. (**f**) After 3 days of ZIKV challenge, RNA levels of ZIKV in mouse sera were analyzed by qRT-PCR analysis. After 8 days of ZIKV challenge, ZIKV RNA levels were measured by qRT-PCR analysis in brain (**g**) and spleen (**h**). **** *p* < 0.0001, ns, not significant, compared with the uninfected group.

The immunogenicity and protective efficacy of the AcHERV-ZIKV against ZIKV infection were assessed in 8-week-old female A129 mice. A129 mice were vaccinated with 4 × 10^7^ FFU of recombinant baculovirus by intramuscular injection, and further boosted at week 3 (Figure 3a). AcHERV-eGFP was used as a negative control. The AcHERV-ZIKV-vaccinated groups showed successful induction of ZIKV-specific IgG and neutralizing antibody (Figure 3b,c). In contrast, the negative control (AcHERV-eGFP) group showed low or no IgG and neutralizing antibody titers after a second immunization. On week 8, vaccinated A129 mice were challenged with 5 × 10^4^ PFU of ZIKV MR766. The vaccinated group was fully protected from lethal ZIKV challenge, and did not show any loss of body weight or clinical sign of illness (Figure 3d,e). In addition, no viremia was detected in these mice after 3 days of ZIKV challenge (Figure 3f). In contrast, all sham-vaccinated mice revealed high viremia at day 3 post-challenge and exhibited a high mortality rate between 6- and 8-days post infection. After mice were sacrificed, viral RNA levels were measured in brain, spleen, and kidney tissues. The sham group showed a high viral RNA copy number, whereas the vaccine group showed a low viral RNA load that was similar to that of uninfected controls (Figure 3g,h). In this experiment, even the uninfected group had several copies of the virus, but the values are the experimental background of qRT-PCR. Among the isolated organs, the brain showed the most considerable difference (~10,000-fold) between the vaccine and negative control groups.

### 3.4. AcHERV-ZIKV Protects Pregnant A129 Mice and Their Fetuses against ZIKV Infection

**Figure 4:**
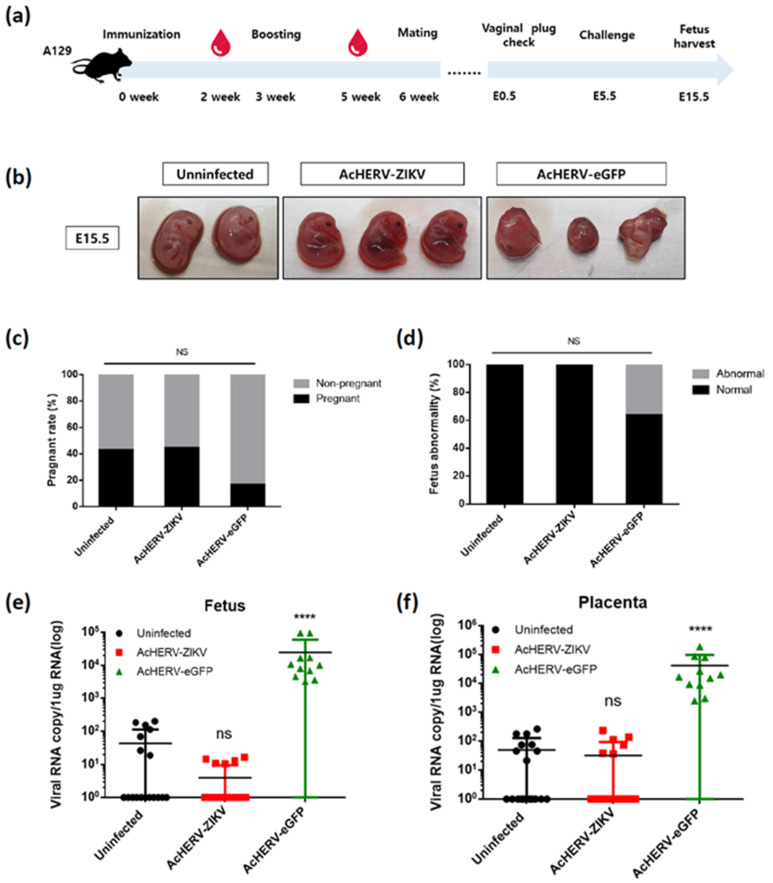
AcHERV-ZIKV protects against placental and fetal ZIKV infection. Female A129 mice (8 weeks old) were immunized with 5 × 10^7^ FFU of AcHERV-ZIKV by intramuscular injection. An AcHERV-eGFP-immunized group was used as a negative control. After 3 weeks, mice were boosted with an equivalent dose of same recombinant baculovirus. Mouse sera were collected at 2 weeks post-immunization. After the presence of a vaginal plug was determined, mice were inoculated with 1 × 10^4^ PFU of ZIKV FLR strain by intraperitoneal injection. (**a**) Schematic illustration of vaccination and virus challenge procedures performed using A129 mice. (**b**) Left panel, normal E15.5 fetuses from uninfected dams. Middle panel, representative images of E15.5 fetuses from AcHERV-ZIKV-vaccinated dams. Right panel, representative images of grossly hylomorphic E15.5 fetuses from AcHERV-eGFP-immunized dams. (**c**) The pregnancy rate in mated and plugged mice (ZIKV, *n* = 9; eGFP, *n* = 18; NTC, *n* = 7). (**d**) The percentage of fetal abnormality (ZIKV, *n* = 18; eGFP, *n* = 11; NTC, *n* = 15). ZIKV loads in the fetuses (**e**) and placentae (**f**) of pregnant mice. **** *p* < 0.0001; ns, not significant, compared with the placebo group.

Eight-week-old female A129 mice were immunized twice at a 3-week interval to evaluate the ability of the virus-based vaccine to protect the fetus during pregnancy. A129 pregnant dams that had been mated with WT C57BL/6 male mice were infected with ZIKV at embryonic days 5.5 (E5.5) and sacrificed at E15.5 (Figure 4a). The date of challenge and infection dose were determined based on previous references. This is because fetuses in early pregnancy (E4.5–5.5) have been reported to be severely affected by ZIKV infection [5,12,23,24].

To minimize the effect of maternal mortality on fetal viability, pregnant mice were inoculated with Asian type ZIKV instead of pathogenic MR766. Individual fetuses were evaluated morphologically for size and appearance (Figure 4b). Not all mice with vaginal plugs had a fetus; the non-infected control and AcHERV-ZIKV-immunized mice had pregnancy rates of 42.86% and 44.44%, respectively (Figure 4c). This is within the normal pregnancy rate of 31–44% recorded for healthy mice by the Jackson Laboratory (USA) [25,26,27]. In contrast, the pregnancy rate of mice vaccinated with AcHERV-eGFP was only 16.67%, which was below the normal range. In addition, 36.34% of the fetuses obtained from the AcHERV-eGFP-treated group showed explicit morphological abnormalities (small size, atypical development, etc.) (Figure 4d). The remaining 63.64% of the fetuses showed relatively normal development but high RNA copy numbers of ZIKV. These observations indicate that the fetuses in the AcHERV-eGFP-treated group were affected by ZIKV infection without the obvious morphological changes. The ZIKV viral copy numbers were undetectable in vaccinated and control fetuses (AcHERV-ZIKV: 45.95; NTC: 86.4), but high in the AcHERV-eGFP treated group (AcHERV-eGFP: 24744.4/average virus copies per 1ug RNA). A similar pattern was observed in placental samples (AcHERV-ZIKV: 100.19; NTC: 110.82; AcHERV-eGFP: 41473.2) (Figure 4e,f). It is known that ZIKV affects the pregnancy rate and induces fetal abnormality in humans. Our results showed that the AcHERV-ZIKV vaccine could protect the fetus from stillbirth or abnormality.

## 4. Discussion

Since the 2015 Brazil outbreak of ZIKV and the discovery of its association with fetal irregularities such as microcephaly and intrauterine growth restriction, ZIKV infection in pregnant women has become a major concern in humans [28]. A major challenge in ZIKV vaccine development has been safety issues. In order to protect the fetus, women of childbearing potential must be immunized before pregnancy, and immunity should provide complete sterile protection [8].

Because ZIKV causes fetal abnormalities and pregnancy cannot be predicted in women, the persistence of antibodies is important for an effective ZIKV vaccine. Baculovirus vectors are non-pathogenic to humans and can be generated simply in a biosafety level 1 facility [29]. Most viral vectors even those that are replication incompetent, still express the vector’s own genes, which causes toxicity problems that should not be overlooked. In contrast, baculoviruses have an absolute safety advantage because they cannot replicate in mammalian cells and rarely express viral genes. One of the limitations of the viral vectors for DNA vaccine is the induction of antibodies against the viral vector itself or the existence of preexisting antibody against the viral vectors, nullifying the boosting effect of the viral vectors after primming [30,31]. Humans do not have pre-existing antibodies nor produce antibodies against the HERV envelope, the AcHERV systems vaccine can function as multiuse delivery systems [32]. The AcHERV system has the advantage of efficiently and safely delivering the target genes from cytoplasm to the nucleus by the nuclear localizing signal of nuclear capsid.

Already, we developed a vaccine platform in baculovirus system designed to express a protein that would induce high levels of intracellular delivery in vitro [22]. Recombinant baculovirus was designed to express eGFP under the control of the CMV promoter, and the HERV envelope gene was inserted downstream of the polyhedrin promoter. GFP positive cells were more abundant in cells infected with AcHERV-eGFP than those infected with Ac-eGFP. FACS and Western blot analysis showed that HERV enveloped baculoviral vector enhanced its gene delivery efficiency (Figure S1).

When ZIKV is infected in humans, IFN-regulated transcriptional activator, signal transducer and activator of transcription 2 (STAT2) is blocked by non-structural protein 5 (NS5), and the IFN signal is suppressed, thereby easily propagating in the host [33]. The primary target for NS5 is human STAT2, thus infection is generally not expected to be achieved in mice [33,34,35]. Recent studies introduced ZIKV infection animal model with IFN-deficiency in AG129 or A129 [36,37]. Interferon (IFN) type I is important for the early immune response and is a major response to protect the host from viral infection [38]. Type I interferon induces an antiviral state in cells to curb viral replication [39]. Secreted type I IFNs bind to the IFNα/β receptor (IFNAR) and activate JAK1 and TYK2 protein kinases [40]. Activated JAK1 and TYK2 subsequently phosphorylate the transcription factors STAT1 and STAT2 resulting in the formation of IFN-stimulated gene factor 3 (ISGF3). ISGF3 binds to IFN-I-stimulated response elements (ISREs) and promotes the transcription of IFN-stimulated genes (ISGs), which antiviral encode proteins [39]. Therefore, STAT2 are the important transcription factors, that mediate IFN signaling and IFN-induced expression of ISGs.

We developed ZIKV vaccine used our baculovirus system and exploited IFN-α/β receptor-deficient mice (A129) in challenge experiments to evaluate the resistance and defense ability against ZIKV. In our challenge study of ZIKV in A129 mice, the AcHERV-ZIKV group showed sterile protection upon two rounds of immunization and maintained a sufficient level anti-ZIKV antibodies for 6 months after the last immunization (Figure 3d,e). Vaccination studies in pregnant female mice were conducted to establish a correlation of immune protection with complete prevention of maternal-fetal transmission of ZIKV. Previous work showed that A129 mice less than 4 weeks old have 100% mortality rate for Asian type ZIKV, which can affect the pregnancy period in fetus; mice aged 4–8 weeks show 60–80% mortality; and mice over 8 weeks of age show 0% mortality [41]. Thus, we used mice aged at least 8 weeks. After two rounds of immunization with AcHERV-ZIKV, all fetuses developed normally compared to the sham (AcHERV-eGFP) group and uninfected group (Figure 4b–d). Overall, vaccination did not reduce the pregnancy rate or cause apparent abnormalities in the fetus or placenta.

In the development of ZIKV vaccines, protective immunity to the fetus has always been classified as a priority [42]. Our present experimental results show that our vaccine provides sufficient protection within or without the pregnancy period and also protected the fetus during pregnancy in mice. Our findings therefore indicate that the AcHERV-ZIKV vaccine could be considered a safe vaccine candidate against ZIKV.

## 5. Conclusions

We have provided evidence that AcHERV can serve as a vaccine platform of Zika virus. We developed a vaccine that delivers the Zika virus prM/E gene using the AcHERV platform system. As a result of the challenge test, it was confirmed that the fetus and mother were completely protected against Zikv infection. AcHERV-ZIKV could be a potential vaccine candidate for human application.

## Figures and Tables

**Table 1:** Signal sequences of different origins used for recombinant baculovirus.

Name	Amino Acid Sequence
ZIKV	MAAEVTRR
CD5	MPMGSLQPLATLYLLGMLVAS
IgK	METDTLLLWVLLLWVPGSTG
IgM	MKFSWVMFFLMAVVTGVNSE

## Data Availability

The data is available from the corresponding author upon request.

## Supplementary Materials

The following are available online at https://www.mdpi.com/article/10.3390/vaccines9050438/s1, Figure S1: Schematic depiction of recombinant baculovirus vectors and the comparison of gene transfer efficiency. Table S1: AcHERV-ZIKV immunization group in C57BL/6 mice.
