# Supplementary material for: The Safe Baculovirus-Based PrM/E DNA Vaccine Protected Fetuses against Zika Virus in A129 Mice
Source: Vaccines (Basel). 2021 Apr 30;9(5):438. doi: 10.3390/vaccines9050438 (PMC8147223; doi:10.3390/vaccines9050438)
Supplement: Supplementary file 1 [file vaccines-09-00438-s001.zip › vaccines-1183892-supplementary.pdf]

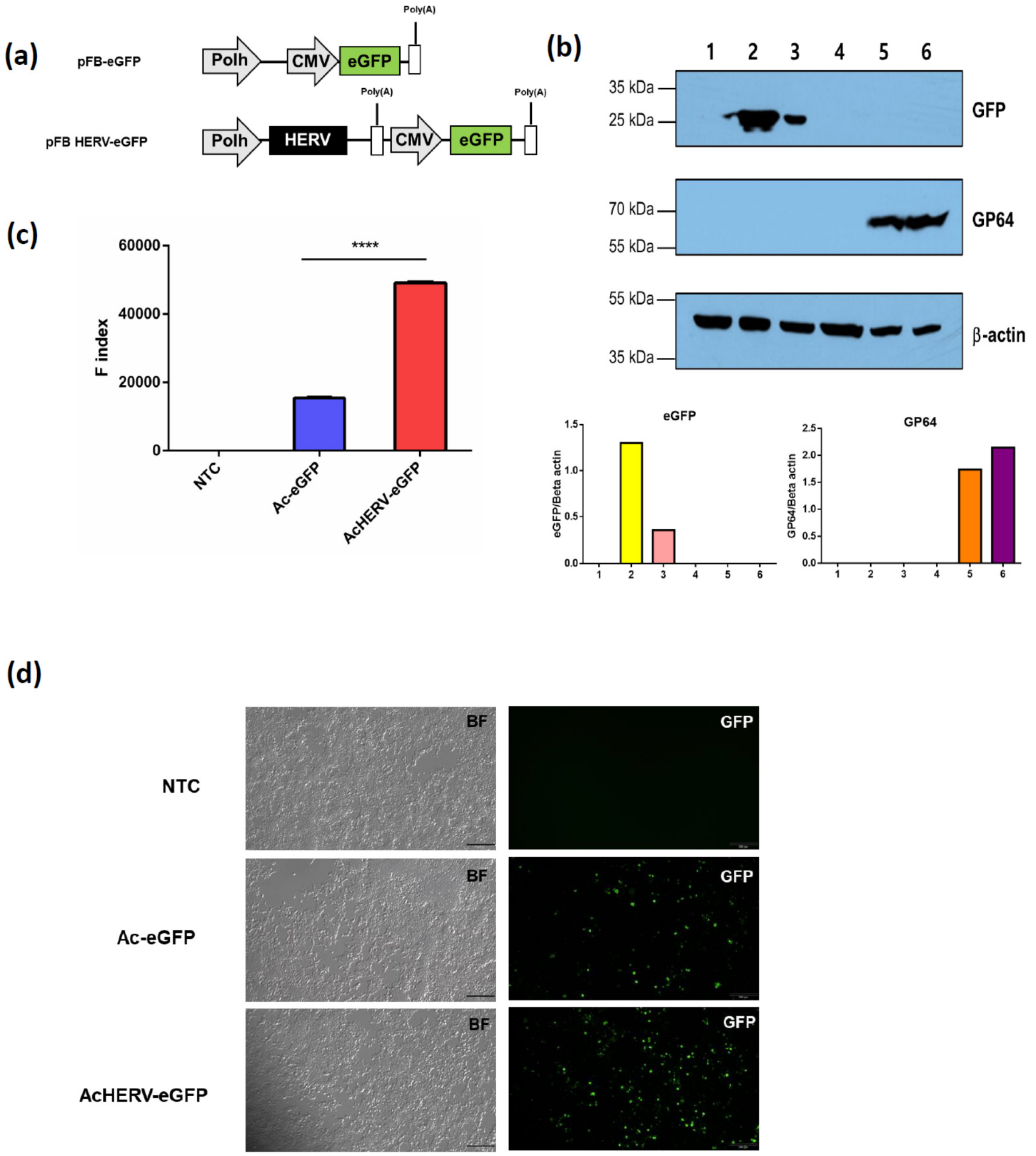

Figure S1. Schematic depiction of recombinant baculovirus vectors and the comparison of gen e transfer efficiency. (a) Diagram of GFP-expressing baculovirus vectors. Both Ac-eGFP and Ac HERV-eGFP express eGFP under the control of the CMV promoter. AcHERV-eGFP expresses t he envelope protein of HERV under the control of the baculovirus polyhedron promoter (Polh). Polh, polyhedron promoter; HERV, HERV envelope protein; CMV, cytomegalovirus promote r; eGFP, enhanced green fluorescence protein. (b) Expression of eGFP and gp64. 293TT cells a nd Sf9 cell were infected with Ac-eGFP, AcHERV-eGFP. Expression levels of prM/E were dete cted by western blot analysis using anti-eGFP antibody and gp64 antibody. ImageJ analysis gr aph of GFP (left) and gp64 (right) protein intensity normalized to β-actin. Lane 1, 293TT; lane 2, AcHERV-eGFP infected 293TT; lane 3, Ac-eGFP infected 293TT; lane 4, Sf9; lane 5, AcHER V-eGFP infected Sf9; lane 6, Ac-eGFP infected Sf9 (c) FACS analysis of recombinant baculovir

us-infected 293TT cells. (F index = GFP positive cells x Geo Mean of GFP). **** $p < 0.0001$ compared with the negative control. (d) Fluorescence images of recombinant baculovirus-infected 293TT cells. GFP-positive cells were more abundant in AcHERV-eGFP-infected cells than in Ac-GFP-infected cells. BR, bright field; NTC, noninfected control.

Table S1. AcHERV-ZIKV immunization group in C57BL/6 mice

Group	Baculovirus	Dose
G1	PBS	100
G2	AcHERV-ZIKV (prM/E△TM with ZIKV SS)	$4X10^7$ FFU
G3	AcHERV-ZIKV (CD5 SS prM/E△TM)	$4X10^7$ FFU
G4	AcHERV-ZIKV (IgK SS prM/E△TM)	$4X10^7$ FFU
G5	AcHERV-ZIKV (IgM SS prM/E△TM)	$4X10^7$ FFU
